# Supplementary material for: A toolbox to engineer the highly productive cyanobacterium Synechococcus sp. PCC 11901
Source: Plant Physiol. 2024 May 7;196(2):1674–90. doi: 10.1093/plphys/kiae261 (PMC11444289; doi:10.1093/plphys/kiae261)
Supplement: kiae261_Supplementary_Data [file kiae261_supplementary_data.zip › 240423 Supplementary Data.pdf]

A toolbox to engineer the highly productive cyanobacterium *Synechococcus* sp. PCC 11901

Authors: Angelo J. Victoria[1,2*], Tiago Toscano Selão[3*], José Ángel Moreno-Cabezuelo[4], Lauren A. Mills[4], Grant A. R. Gale[1,2], David J. Lea-Smith[4], Alistair J. McCormick[†,1,2]

[1] Institute of Molecular Plant Sciences, School of Biological Sciences, University of Edinburgh, EH9 3BF, UK.

[2] Centre for Engineering Biology, School of Biological Sciences, University of Edinburgh, EH9 3BF, UK.

[3] Department of Chemical and Environmental Engineering, University of Nottingham, Nottingham NG7 2RD, UK.

[4] School of Biological Sciences, University of East Anglia, Norwich Research Park, Norwich, NR4 7TJ, UK.

SUPPLEMENTARY DATA

Supplementary Table S1. Table of all CyanoGate-compatible parts generated in this work.

Supplementary Table S2. All plasmid vectors made and used in this study. See 'Supplementary Table S2 - Plasmids (all).xlsx'.

Supplementary Table S3. Primer and sgRNA oligonucleotides used in this study. See 'Supplementary Table S3 - Primer and sgRNA oligonucleotides.xlsx'.

Supplementary Data S1. Sequence maps (.gb files) for plasmid vectors in Supplementary Table S1. See 'Supplementary Data S1.zip'.

Supplementary Figure S1. Analysis of putative Gcn5-related N-acetyltransferase (GNAT) family genes in *Synechococcus* sp. PCC 11901.

Supplementary Figure S2. Representative PCR-based segregation analysis of *Synechococcus* sp. PCC 11901 transformants targeting neutral sites *glgA1* and *aquI*.

Supplementary Figure S3. Self-replicating plasmid stability in *Synechococcus* sp. PCC 11901 transconjugants.

Supplementary Figure S4. Growth of *Synechococcus* sp. PCC 11901 with varying doses of small molecule inducers.

Supplementary Figure S5. Growth analysis of CRISPRi-dCas9 strains targeting eYFP.

Supplementary Figure S6. Analysis of CRISPRi-dCAs9 strains targeting *cpcB* and *nblA*.

Supplementary Figure S7. Attempted generation of markerless mutants using the CRE-Lox system.

Supplementary Figure S8. CRISPR-Cas12a double HR editing approach – sgRNA, repair template and hybrid suicide vector assembly.

Supplementary Figure S9. Pipeline for iterative CRISPR-Cas12a editing using the double HR approach.

Supplementary Figure S10. Confirmation of recombination between the hybrid suicide vector and editing vector.

Supplementary Table S1. Table of all new CyanoGate-compatible parts generated in this work.

No.	Vector ID	Part name	Level	5' overhang	3' overhang	Backbone	Domesticated	Selection	Notes	References
**Acceptors**										
**Level T**										
1	pCAT.482	pPMQSK1-T	T	TGCC	GGGA	pPMQSK	yes	SpR/KmR	Modified BioBrick vector, RSF1010-derived ori.	Huang et al., 2010
**Level 1**										
1	pCA1.339	pPMQSK1-1	1	GGAG	GGGA	pPMQSK	yes	SpR/KmR	Modified BioBrick vector, RSF1010-derived ori.	Huang et al., 2010
**Level 0**										
1	pCA0.421	sgRNA acceptor	0	AATG	AGGT	pICH41258	no	SpR	Acceptor of sgRNA(s) for CRISPR-Cas12a gene editing	this study
**Insertion Sites**										
1	pC0.378	*desB* Down Flank	0	GCTT	CGCT	pICH41276	no	SpR	*Synechococcus* sp. PCC 11901 sequence downstream of FEK30_04840.	Mills et al., 2022 Vogel et al., 2017
2	pC0.385	*desB* Up Flank	0	GGAG	AATG	pICH41295	yes	SpR	*Synechococcus* sp. PCC 11901 sequence upstream of FEK30_04840.	Mills et al., 2022 Vogel et al., 2017
3	pC0.386	*glgA1* Up Flank	0	GGAG	AATG	pICH41295	no	SpR	*Synechococcus* sp. PCC 11901 sequence upstream of FEK30_14880.	Sengupta et al., 2020, Mittermair et al., 2021
4	pC0.387	*glgA1* Down Flank	0	GCTT	CGCT	pICH41276	no	SpR	*Synechococcus* sp. PCC 11901 sequence downstream of FEK30_14880.	Sengupta et al., 2020, Mittermair et al., 2021
5	pC0.388	*mrr* Up Flank	0	GGAG	AATG	pICH41295	yes	SpR	*Synechococcus* sp. PCC 11901 sequence upstream of FEK30_09380.	this study
6	pC0.389	*mrr* Down Flank	0	GCTT	CGCT	pICH41276	yes	SpR	*Synechococcus* sp. PCC 11901 sequence downstream of FEK30_09380.	this study
7	pC0.419	*aquI* Down Flank	0	GCTT	CGCT	pICH41276	no	SpR	*Synechococcus* sp. PCC 11901 sequence downstream of FEK30_10065.	this study
8	pC0.420	*aquI* Up Flank	0	GGAG	AATG	pICH41295	no	SpR	*Synechococcus* sp. PCC 11901 sequence upstream of FEK30_10065.	this study
9	pC0.425	RSF1010 Up Flank	0	GGAG	AATG	pICH41295	no	SpR	RSF1010 upstream homology sequence	this study

10	pC0.426	RSF1010 Down Flank	0	GCTT	CGCT	pICH41276	no	SpR	RSF1010 downstream homology sequence	this study
11	pC0.437	NS1 Up Flank	0	GGAG	AATG	pICH41295	no	SpR	*Synechococcus* sp. PCC 11901 sequence upstream of an intergenic region of 185bp between FEK30_11550 and FEK30_11555)	Bernhards et al., 2022
12	pC0.438	NS1 Down Flank	0	GCTT	CGCT	pICH41276	yes	SpR	*Synechococcus* sp. PCC 11901 sequence downstream of an intergenic region of 185bp between FEK30_11550 and FEK30_11555)	Bernhards et al., 2022
**Promoters**										
1	pC0 422	P_{rhaBAD}	0	GGAG	AATG	pICH41295	no	SpR	L-rhamnose inducible promoter	Kelly et al., 2018
2	pC0.423	P_{phlF}	0	GGAG	AATG	pICH41295	no	SpR	2,4-diacetylphloroglucinol inducible promoter from *Pseudomonas fluorescens*	Meyer et al., 2019
3	pC0.394	P_{trcE*}	0	GGAG	AATG	pSB4K5	no	SpR	theophylline responsive riboswitch E*	Nakahira et al., 2014
**Parts**										
1	pC0.393	Ab^{R}Gent	0	AGGT	GCTT	pICH41264	no	SpR/ GmR	Gentamicin resistance cassette	this study
2	pC0.391	Cas12a (FnCpf1)	0	AATG	GCTT	pICH41308	no	SpR	Cas12a/Cpf1 from Francisella novicida	Zetsche et al., 2015
**Other**										
1	pC1.509	Cas12a editing vector	1			pPMQSK1-1	no	SpR	Editing vector for CRISPR-Cas12a gene editing (see Figure 6 for details)	this study
2	pC1.530	Curing vector (GmR) for editing strain	1			pPMQSK1-1	no	SpR	Curing vector with sgRNA and GmR for removing Cas12a editing vector (see Figure S9 for details)	this study

Additional references

Zetsche, B., Gootenberg, J. S., Abudayyeh, O. O., Slaymaker, I. M., Makarova, K. S., Essletzbichler, P., Volz, S. E., Joung, J., van der Oost, J., Regev, A., Koonin, E. V., & Zhang, F. (2015). Cpf1 Is a Single RNA-Guided Endonuclease of a Class 2 CRISPR-Cas System. *Cell*, *163*(3), 759–771. https://doi.org/10.1016/j.cell.2015.09.038

Supplementary Table S2. All plasmid vectors made and used in this study. See 'Supplementary Table S2 Plasmid vectors (all).xlsx'. RSF1010 vectors used for promoter and terminator characterizations in Figure 3B and 3C, respectively, have been published previously (Vasudevan et al., 2019; Gale et al., 2021).

Supplementary Table S3. Primer and sgRNA oligonucleotides used in this study. See 'Supplementary Table S3 - Primer and sgRNA oligonucleotides.xlsx'.

Supplementary Data S1. Sequence maps (.gb files) for plasmid vectors in Supplementary Table S1. See 'Supplementary Data S1.zip'.

A

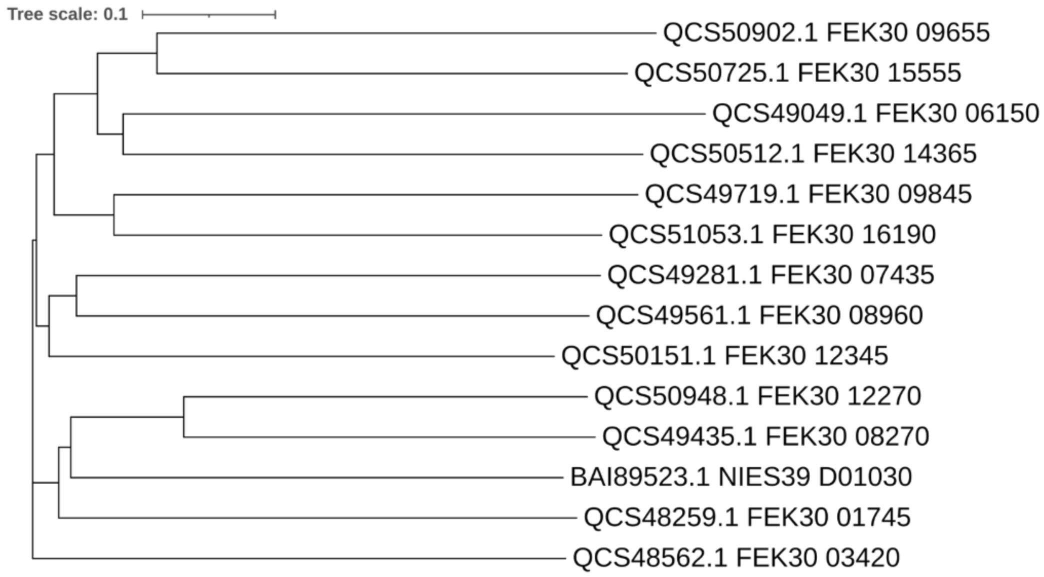

B

```
Aligned_sequences: 2
# 1: BAI89523.1
# 2: QCS49435.1
# Matrix: EBLOSUM62
# Gap_penalty: 10.0
# Extend_penalty: 0.5
# Length: 219
# Identity:      46/219 (21.0%)
# Similarity:    68/219 (31.1%)
# Gaps:          87/219 (39.7%)
# Score: 100.0

CLUSTAL O(1.2.4) multiple sequence alignment

BAI89523.1          MRIINLSPNNQNHIHQAATLLV-AEFRENWPNAWPTYERGLAEVMESFGDDRVNLVAVDE   59
QCS49435.1          MNKISDCSNILFSVDKARVDLVQLQALFNATAFWA-RERSLADLETAIA-YSDPVVTVWD   58
                    *. *. . *    :.:* . **  :   *    *   **.**::  ::.     :*:* :

BAI89523.1          NDNLLGWIGGIS--QYQGHVWELHPLVVKSDYQGLGIGRKLVANLEDYVRSQGGLTLWLG   117
QCS49435.1          GDRLIGFTRGTSDGVFRATVWD---VVIHPDYQGLGLGRKLVETLISHPRMCRVERVYLM   115
                    .*.*:*:  * *   ::. **:   :*:: ******:***** .* .: *      ::*

BAI89523.1          TDDENNLTSLSGVELYPHFLENIANIKNHGRHPYEFYQKCGFVIMGVVPDANGIGKPDIL   177
QCS49435.1          TTH-----------------------------QQTFYERIGFKENATTTM----------   136
                    * .                                  **:: **   ...

BAI89523.1          MAKSLRIDNHPKSN-------------      191
QCS49435.1          ---VLHNHEHPISVITCAVEETAPLPA      160
                       *: .:** *
```

Supplementary Figure S1. Analysis of putative Gcn5-related N-acetyltransferase (GNAT) family genes in *Synechococcus* sp. PCC 11901. **(A)** Phylogenetic tree of 13 GNAT family proteins in PCC 11901 and the aminoglycoside acetyltransferase from *Arthrospira platensis*

(BAI89523.1) (built by Clustal Omega (EMBL-EBI)). **(B)** Sequence alignment and scores between BAI89523.1 and a putative aminoglycoside acetyltransferase from PCC 11901 (QCS49435.1). The conserved coenzyme A binding pocket is highlighted in yellow. The alignment was performed in EMBOSS Needle.

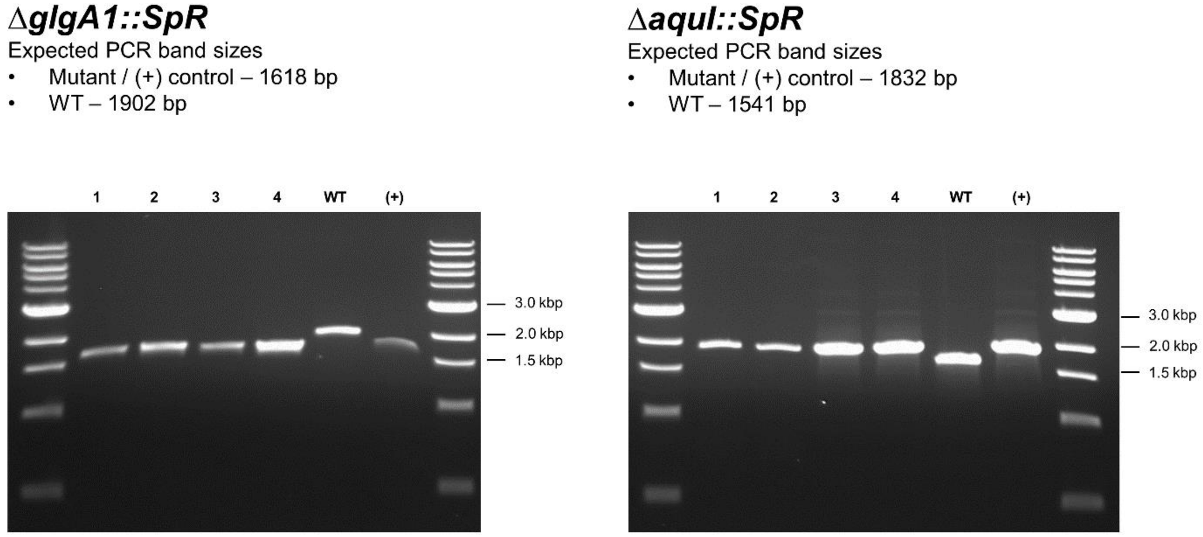

Supplementary Figure S2. Representative PCR-based segregation analysis of *Synechococcus* sp. PCC 11901 transformants targeting neutral sites *glgA1* and *aquI*. Electrophoresis gel profiles are shown for four independent transformant colonies after one round of re-streaking on selective agar. Full segregation for each transformant is demonstrated by the presence of amplicons showing insertion of the SpR cassette at the *glgA1* (1,618 bp) and *aquI* (1,832 bp) loci and the absence of a respective wild-type (WT) amplicon (1,902 bp for *glgA1* and 1,541 bp for *aquI*). Amplicons from each transformation plasmid vector (+) are included as positive controls.

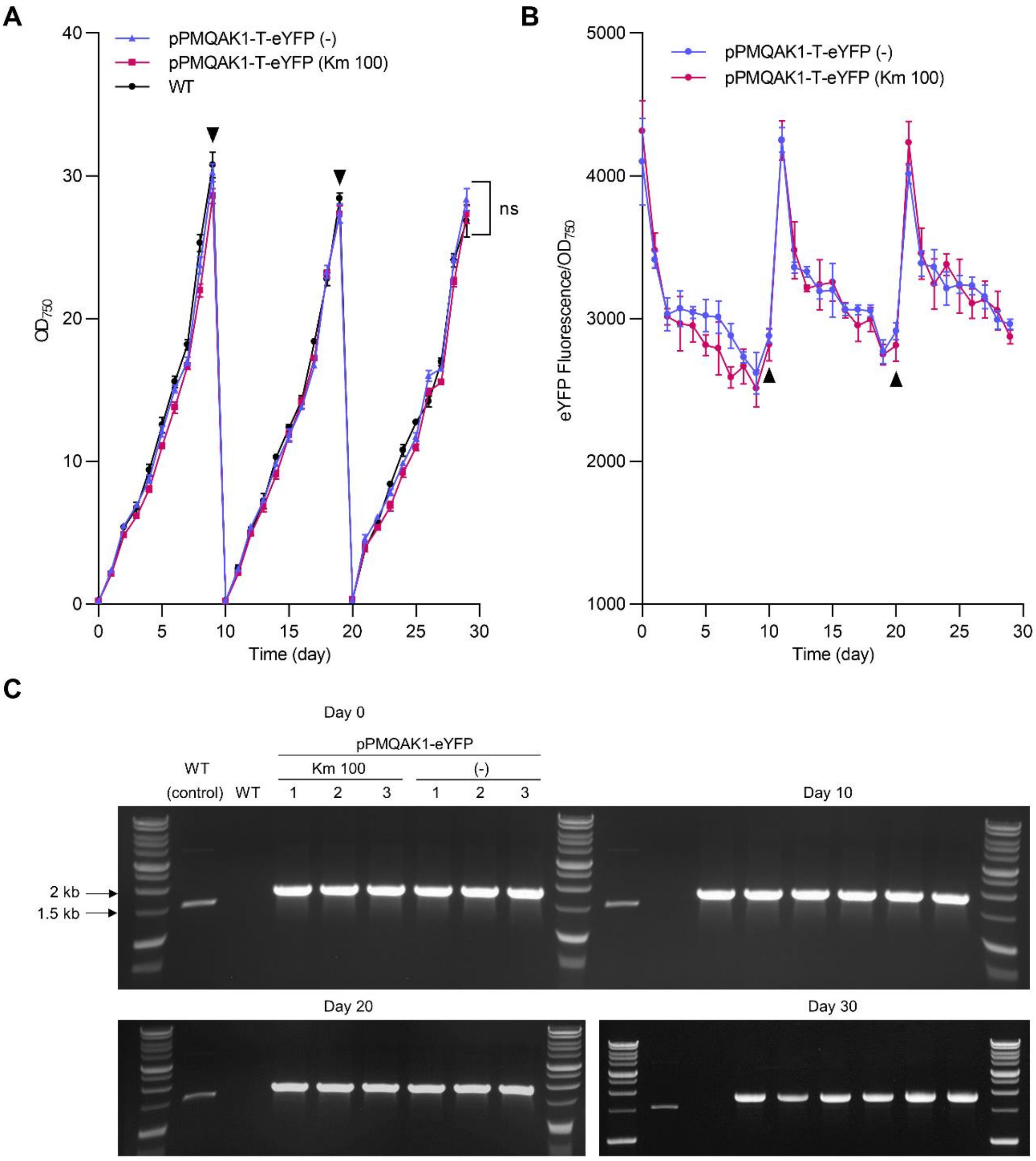

Supplementary Figure S3. Self-replicating plasmid stability in *Synechococcus* sp. PCC 11901 transconjugants. (**A**) Growth of WT and transconjugant (pPMQAK1-T-eYFP) cultures in the presence (Km 100, 100 µg mL^{-1}) or absence (-) of kanamycin over a 30-day growth period. (**B**) Normalized eYFP fluorescence of the pPMQAK1-T-eYFP conjugant in the presence or absence of kanamycin. Black arrowheads indicate timepoints when cultures were re-diluted to OD_{750}=0.2. Error bars represent mean ±SEM of three biological replicates. No significant differences (ns) in growth were found on day 30 ($P < 0.05$) as determined by ANOVA followed by Tukey's honestly significantly different test. **(C)** Verification of the presence of the pPMQAK1-T-eYFP self-replicating vector by PCR of samples taken at days 0, 10, 20, and 30. Bands indicate the presence (1,801 bp) or absence of the pPMQAK-1-T-eYFP vector. The aquI locus was amplified from WT DNA as an additional control (1,541 bp).

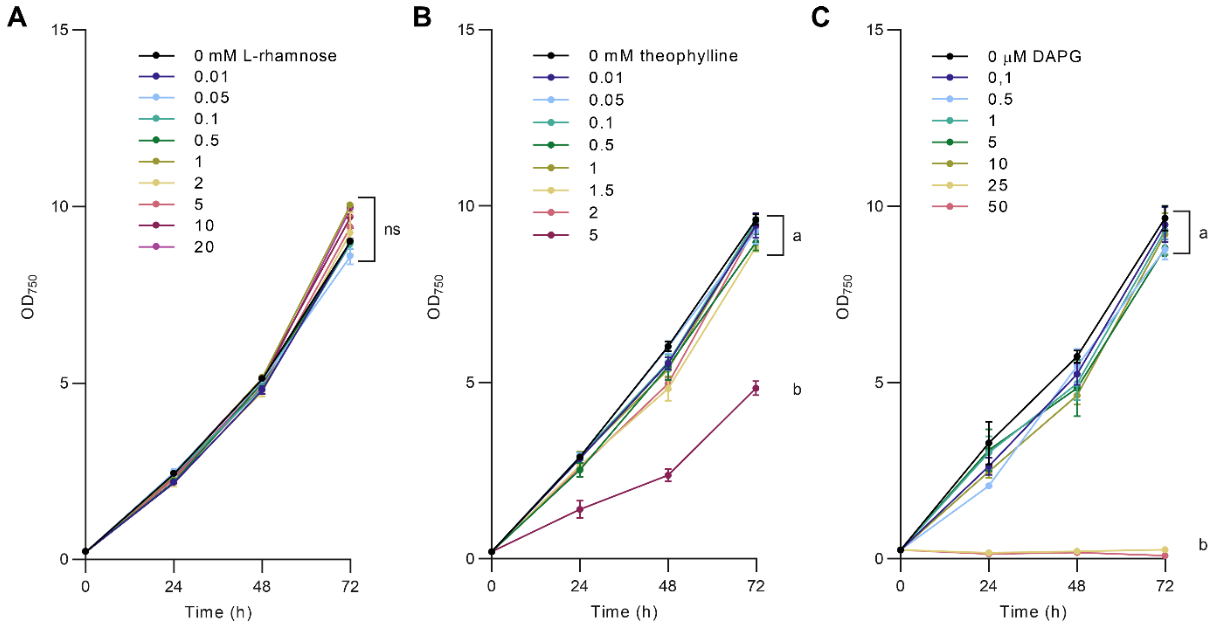

Supplementary Figure S4. Growth of *Synechococcus* sp. PCC 11901 with varying doses of small molecule inducers. Growth of wild-type PCC 11901 in increasing concentrations of **(A)** L-rhamnose, **(B)** theophylline, and **(C)** DAPG over 72 h. Error bars represent the mean ±SEM of three biological replicates. No significant difference (ns) and lowercase letters indicating significant difference ($P < 0.05$) are shown, as determined by ANOVA followed by Tukey's honestly significant difference tests.

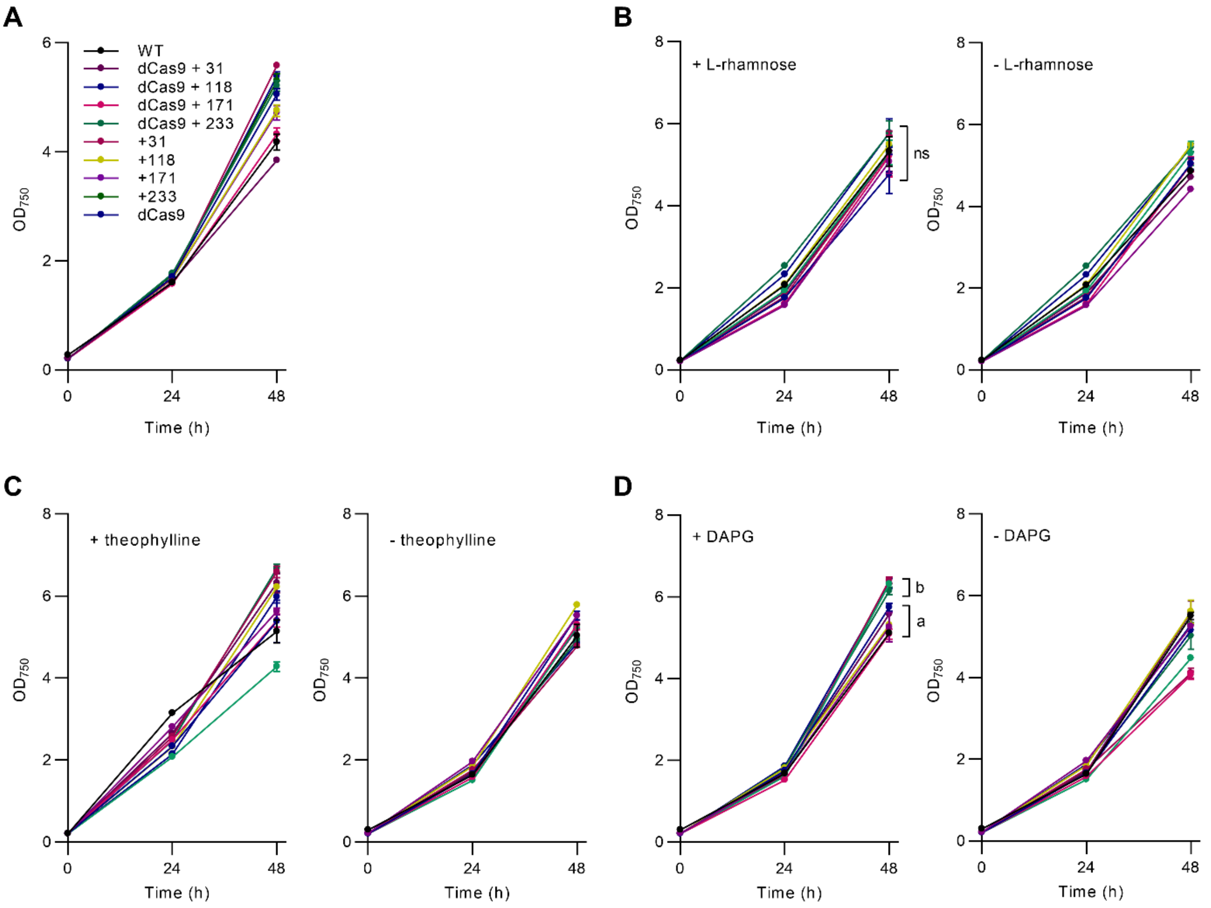

Supplementary Figure S5. Growth analysis of CRISPRi-dCas9 strains targeting eYFP. **(A)** Growth of P_{J23113} CRISPRi strains in MAD medium. **(B)** Growth of RhaS/P_{rhaBAD} CRISPRi strains in the presence or absence of 10 mM L-rhamnose. **(C)** Growth of $P_{\mathit{trcE*}}$ CRISPRi strains in the presence or absence of 2 mM theophylline. **(D)** Growth of PhlF/P_{phlF} CRISPRi strains in the presence or absence of 10 µM DAPG. Error bars represent mean ±SEM of three biological replicates. No significant difference (ns) and lowercase letters indicating significant difference ($P < 0.05$) are shown, as determined by ANOVA followed by Tukey's honestly significant difference tests.

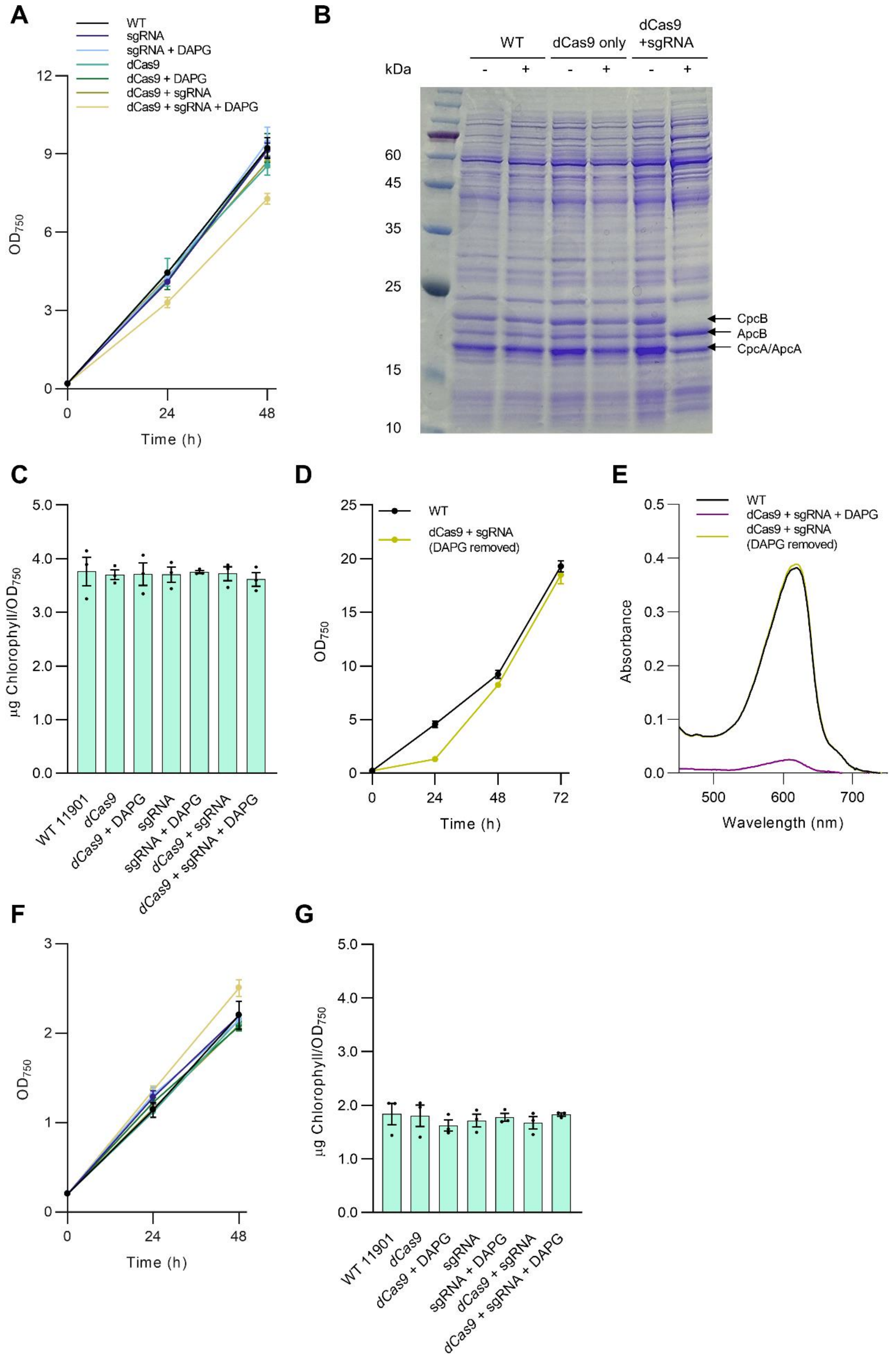

Supplementary Figure S6. Analysis of CRISPRi-dCAs9 strains targeting *cpcB* and *nblA*. (**A**) Growth of the *cpcB* CRISPRi strains with and without inducer (10 µM DAPG) over 48 h (from Figure 5F and 5G). **(B)** Coomassie-stained SDS-PAGE gel of PBS extracts from the *cpcB*

CRISPRi strain carrying both sgRNA and dCas9 in the absence (-) or presence (+) of DAPG. Arrows indicate the bands for ApcB (18.7 kdA), CpcB (18.1 kdA), CpcA (17.6 kdA) and ApcA (17.3 kdA). **(C)** Chlorophyll contents of the *cpcB* CRISPRi strains. **(D)** Growth of the *cpcB* CRISPRi strain carrying both sgRNA and dCas9 in comparison to wild-type PCC 11901 following removal of DAPG. **(E)** Absorbance spectra of PBS extracts following removal of DAPG at 48 h. (**F**) Growth of the *nblA* CRISPRi strains with and without inducer over 48 h. **(G)** Chlorophyll contents of *nblA* CRISPRi strains. Error bars show the mean ±SEM of three biological replicates.

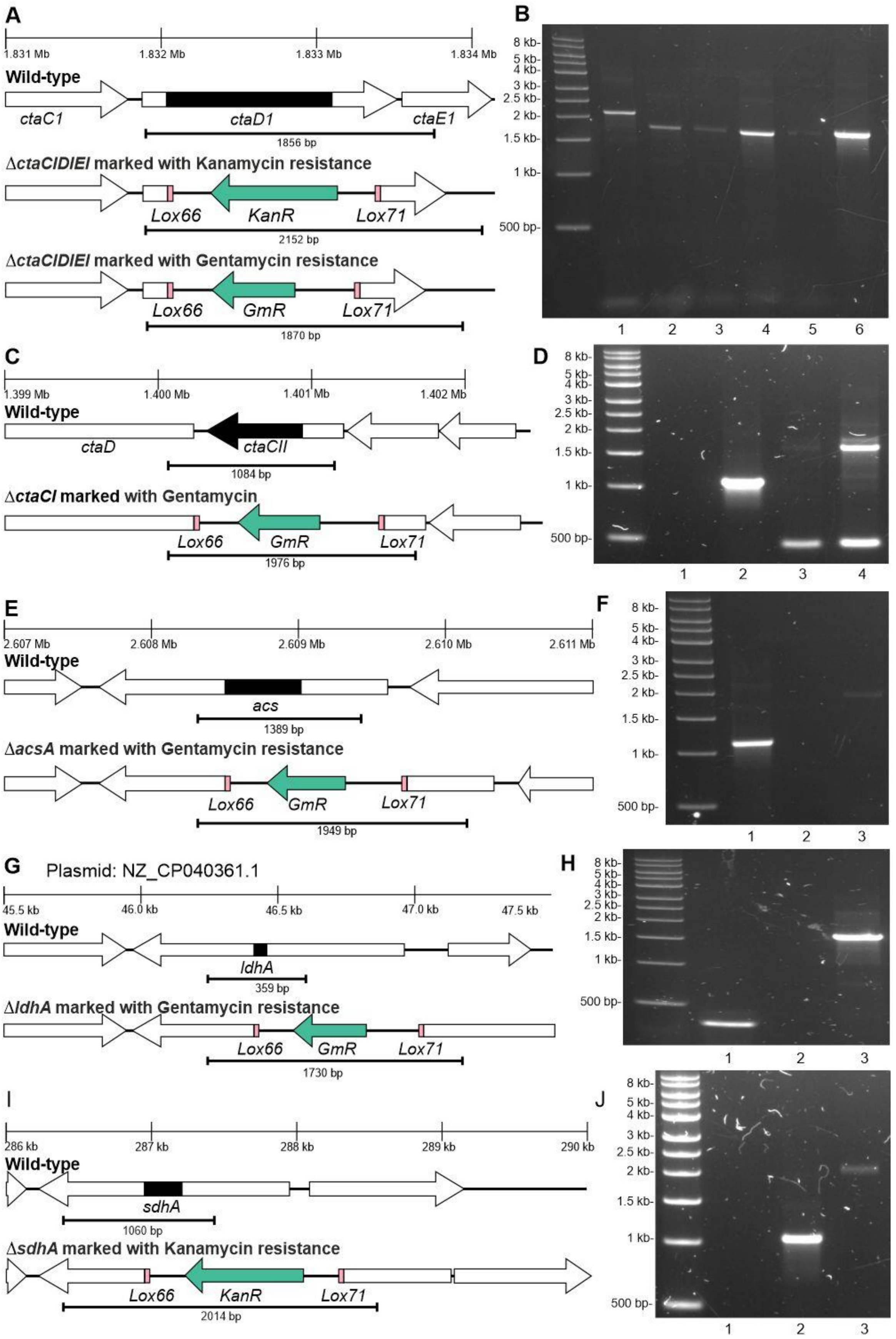

Supplementary Figure S7. Attempted generation of markerless mutants using the CRE-Lox system. Schematic representations of locus location in the PCC 11901 genome (top) and the

wild-type (middle) and marked knockout (KO, bottom) profiles expected in the **(A)** *ctaDIEI*, **(C)** *ctaCII*, **(E)** *acs*, **(G)** *ldhA* and **(I)** *sdhA* strains following amplification with primers flanking the deleted sequence. Regions deleted in the mutant strains are shaded in black. Amplification of genomic DNA in: **(B)** Δ*ctaDIEI* KmR marked KO (lane 1); Δ*ctaDIEI* GmR marked KO (lanes 2-5); WT (lane 6); **(D)** negative control (lane 1); WT (lane 2); Δ*ctaCII* GmR marked KO (lanes 3-4); **(F)** WT (lane 1); negative control (lane 2); Δ*acs* GmR marked KO (lane 3); **(H)** WT (lane 1); negative control (lane 2); Δ*ldhA* GmR marked KO (lane 3); **(J)** negative control (lane 1); WT (lane 2); Δ*sdhA* KmR marked KO (lane 3). Δ*ctaDIEI* GmR marked KOs were sequenced to confirm the correct KO profile.

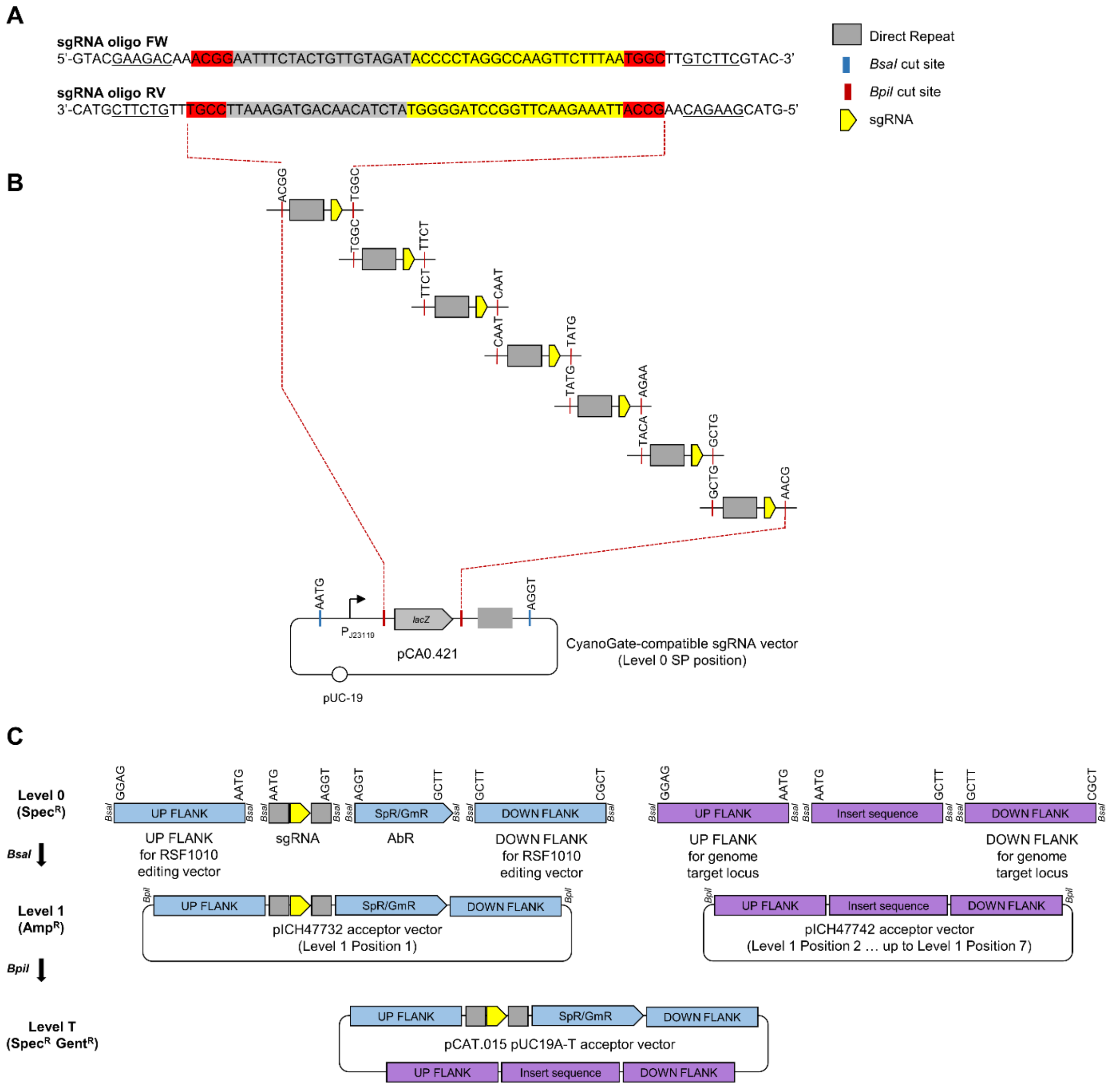

Assembly of sgRNA plasmid vectors (adapted from Liao et al., 2019)

1. To anneal oligonucleotides for making dsDNA parts for assembly (A), add 15 µL of the forward and reverse oligonucleotides into a PCR tube and incubate in a thermal cycler using the following step-down program: 95°C for 5 min, then decrease by 1°C incrementally and hold for 30 seconds for 79 cycles (until 25°C).
2. Level 0 assembly (B): In a PCR tube, add 2 µL of T4 ligation buffer, 2 µL of ATP (10 mM), 1 µL of each annealed repeat-spacer subunit (from 1.), 50 ng of the sgRNA acceptor vector (pCA0.421, Supplementary Table S1), 0.5 µL of T4 ligase, 1.5 µL of BpiI, and water to a total volume of 20 µL. Mix by pipetting and spin down briefly. Incubate the tube in a thermal cycler using the following program: 30 cycles of alternating digestion and ligation (37°C for 5 min, 16°C for 5 min) followed by a final digestion step (60°C for 5 min).
3. In a 1.5 mL microcentrifuge tube, transform 10 µL of the assembly reaction into 30 µl of competent TOP10 *E. coli* cells using the heat shock method. Add 500 µL LB to the cells and shake at 37°C for 1 h. Plate the cells in LB gar supplemented with 100 ug/mL spectinomycin and 40 ug/mL X-gal, and incubate overnight at 37 °C.
4. Screen the white colonies by colony PCR and Sanger sequencing.

Supplementary Figure S8. CRISPR-Cas12a double HR editing approach – sgRNA, repair template and hybrid suicide vector assembly. **(A)** sgRNA were assembled by annealing oligonucleotides incorporating the desired 18-22bp sgRNA (yellow), direct repeat (grey),

unique 4 bp overhangs (red) and BpiI sites (underlined) for **(B)** assembly into the pC0.421 sgRNA acceptor vector. Overhangs match those from the CRATES system and allow for assembly of an array of between one and seven sgRNAs (Liao et al., 2019). **(C)** MoClo assembly of level 0 parts into level 1 vectors incorporating the sgRNA(s) in position 1 and homology repair template(s) in position 2 up to position 7 using the available Plant MoClo level 1 acceptor vectors (Engler et al. 2014). The level 1 vectors are then assembled together into the level T pCAT.015 pUC19A-T acceptor vector (Vasudevan et al., 2019). Using this system between one and six genomic loci can be targeted simultaneously. A protocol for assembly of the sgRNA plasmid vectors is included.

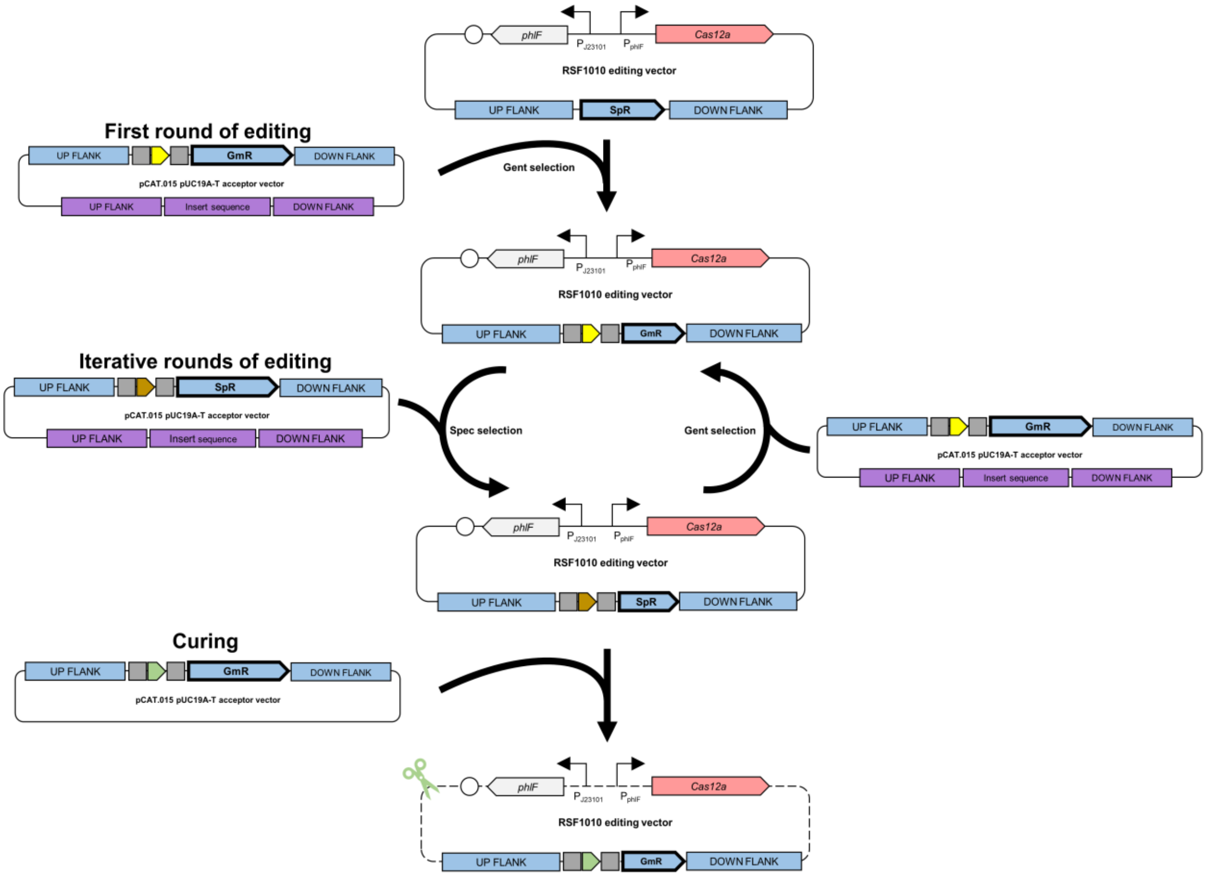

Protocol for transformation with hybrid suicide vectors

1. Grow the editing strain from OD_{750} = 0.2 to the initial stages of exponential growth (OD_{750} = ~0.8).
2. Centrifuge 1 mL of the editing strain for 10 min at 3,500 *g* and wash the cell pellet with fresh culture media three times to remove antibiotics.
3. Dilute the washed editing strain to OD_{750} = 0.2 and add 1.5 µg of purified hybrid suicide vector to 1 mL of the editing strain.
4. Incubate for 4 h at 30°C in 2% (v/v) CO_2 under warm white LED light (150 µmol photons m^{-2} s^{-1}) before adding 10 µM DAPG and continuing incubation overnight.
5. Plate the transformation mixture on to agar plates supplemented with appropriate antibiotics (25 µg mL^{-1} spectinomycin, 50 µg mL^{-1} gentamicin) and 10 µM DAPG and incubate as described in step 4.
6. Screen for mutant colonies using colony PCR.

Supplementary Figure S9. Pipeline for iterative CRISPR-Cas12a editing using the double HR approach. In the first round of editing the hybrid suicide vector recombines with the editing vector (see **Supplementary Figure S8**) to introduce an sgRNA (or sgRNA array) and replaces the SpR with GmR for subsequent selection on gentamicin-supplemented agar plates (also see **Figure 6A**). Subsequent rounds of editing involved cycling between hybrid suicide vectors that exchange SpR and GmR for delivery of new sgRNA(s) to the editing vector. Curing the edited strain of the editing vector to generate a fully markerless strain is done using a hybrid suicide vector (e.g. pC1.530, **Supplementary Table S1**), which delivers a self-targeting sgRNA to the

editing vector. A protocol for transformation with the hybrid suicide vector and induction with DAPG is included.

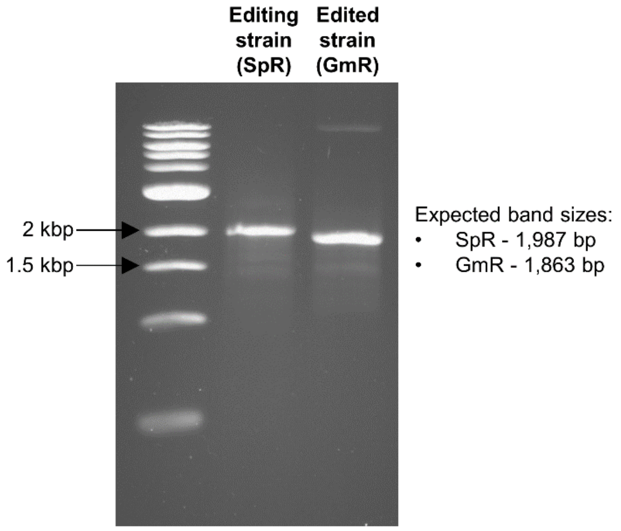

Supplementary Figure S10. Confirmation of recombination between the hybrid suicide vector and editing vector. Example colony PCR of a transformant showing replacement of SpR by GmR.
