# Supplementary material for: A toolbox to engineer the highly productive cyanobacterium Synechococcus sp. PCC 11901
Source: Plant Physiol. 2024 May 7;196(2):1674–90. doi: 10.1093/plphys/kiae261 (PMC11444289; doi:10.1093/plphys/kiae261)
Supplement: kiae261_Supplementary_Data [file pp.00507.2024-s06.pdf › pp.00507.2024-s06.pdf]

Plant Physiology®

A toolbox to engineer the highly productive cyanobacterium Synechococcus sp. PCC 11901

Angelo Victoria, Tiago Selão, José Moreno-Cabezuelo, Lauren Mills, Grant Gale, David Lea-Smith, and Alistair McCormick

Review Timeline:

Submission Date:	04-Aug-2023
Editorial Decision:	16-Oct-2023
Resubmission Received:	09-Apr-2024
Accepted:	14-Apr-2024

16-Oct-2023

Prof. Alistair J McCormick
The University of Edinburgh School of Biological Sciences
School of Biological Sciences
Edinburgh EH9 3BF
United Kingdom

RE: Engineering the highly productive cyanobacterium Synechococcus sp. PCC 11901

Dear Dr. McCormick:

Thank you for giving us the opportunity to review your manuscript for publication in Plant Physiology. The reviewers have found the manuscript of interest, but have raised a number of concerns that will require your attention. As conveyed in the transferred comments below, you will generally find that the reviewers express enthusiasm for your group's work and the number of additional tools that have been developed for this non-model cyanobacterium. I have independently reviewed the manuscript and agree with this assessment.

The reviewers have noted a number of potential areas where the manuscript could be further strengthened. The majority of this feedback relates to questions of logic, language, or requests for additional context. A couple of reviewer comments relate to the strength of conclusions drawn and may benefit from additional supporting data - such as the query raised about supporting data to distinguish CRISPRi from off-target phenotypes (see Reviewer #2). I wish to also direct your attention to the annotated file supplied by Reviewer #2 that should be attached to this message, as it may help you in contextualizing some of their comments.

Because the collective number of comments/queries from the reviewers will likely require substantial revisions to your manuscript, you may require additional time to address them. I am therefore rejecting the manuscript in its present form - but wish to reiterate that there is enthusiasm for the work described in this manuscript. Therefore, I strongly encourage your team to resubmit a revised manuscript that incorporates your responses.

If you do have any queries or concerns, you are welcome to contact me in the first instance.

Thank you again for allowing Plant Physiology to review your work. We look forward to hearing from you soon.

Sincerely,

Board of Editors Plant Physiology

-- Reviewer comments:
Reviewer #1 (Comments for the Author):

Comments for the paper titled "Engineering the highly productive cyanobacterium Synechococcus sp. PCC 11901"
In the research article under review, authors describe the toolbox development for one of the recent, high biomass generating, marine cyanobacterial strain Synechococcus sp. PCC 11901. The authors have performed detailed characterization of different promoters, their regulation, antibiotic resistances, neutral sites, etc. for leveraging this system as a platform cell factory for production of different chemicals. While the article meticulously describes about these developments and present a number of helpful findings, there are some necessary revisions before considering for the publication.
Comments:

1. Title: The manuscript title is very broad, whereas the paper is specifically directed towards toolbox development. Authors can rephrase the title to specifically convey the content of the paper.
2. Neutral sits for
3. Introduction:
a) Authors can try to refer to more recent publications (2020-2023) and be precise with the same. For instance, line 52-53 in the introduction mentions about PCC 6803 and PCC 7942; but the first reference for biofuels (Kopka et al., 2017) is based on PCC 7002. Authors can either include PCC 7002 as an organism and/ or include references, https://doi.org/10.1002/bit.28479, https://doi.org/10.1016/j.biortech.2021.125852.
b) Line 58: When authors mention slow growth rate, it will be good to mention '...as compared to...', because cyanobacteria have faster growth rate among photoautotrophs, but with less biomass generation.
c) Line 73: Authors refer to the cell densities of E coli as 24.7 g/l. This needs correction and a more appropriate reference. Cell densities of 100-150 g DCW/lit are routinely obtained for E coli.
d) High cell densities for cyanobacteria have been reported previously as well. E.g. Alagesan et al 2013 Bioresource Technology

e) Lines 142-143: Additional neutral sites have been reported by PCC 11801 by Madhu et al 2023 ACS Synthetic Biol. Comparison to those may be useful.
4. Results and discussion:
a) I did not find any mention about how stable the transformed cultures are. Metabolically engineered strains of cyanobacteria are notoriously unstable and tend to revert.
b) Authors need to check the entire manuscript for any typo-errors. For instance, line 117 has PC11901 instead of PCC 11901.
c) Figures:
a) In figure 1, giving the pictures of the colonies on the plates look feasible, but does not convey any significant results to the reader. Authors can think of moving it to the supplementary information or remove it completely.
b) In figure 1, the term 'vector' is used in the first column, while the third column indicates transformants per ug of 'plasmid'. Although the two jargons can be used interchangeably, they are not the same. So it would be good to be consistent.
c) There are multiple sub-figures in figures 2, 3, 5, 6 that lack the X-axis label. E.g. 2F, 3A/B/D, 4B/C/D/E, etc.
d) Authors have separately labelled each sub-figure within figure 5. It would be good to club certain figures, because independently, they are unable to convey any result. E.g. F-G, J-K, etc. Secondly, the figure legends mentioned in H, are assumed to be applicable for everything thereafter. However, it needs to be specifically mentioned in the caption or presented as a common legend at the bottom of the four figures.

Reviewer #2 (Comments for the Author):

The manuscript focus on identifying and characterising genetic tools for a cyanobacterium that is fast growing and of interest as a host for biotechnological applications. There is a lack of synthetic biology parts and tools for engineering this photosynthetic microbe efficiently. To this reviewer the objective of this manuscript is dual, first to characterise, analyse and evaluate different tools and second to recommend a valuable tool-box. Maybe the manuscript would benefit from being more distinct about when either is discussed. The work presented are of high quality and a number of useful tools have been developed and characterized.
The manuscript is very clearly written and the figures are informative with smart design and add to the manuscript. The work is sound and all methods and strategies used are in general appropriate for the study. Novel and potentially very useful results are presented. We have a few major concerns with this manuscript. These are communicated to the authors and should be addressed. Some additional experiments are strongly suggested for the work to be as useful as stated in the conclusion.

RESULT AND DISCUSSION
General: The Result and Discussion part is overwhelming, maybe too detailed and some of the important results are masked by much less important information/text. A more focused R&D would be beneficial and a short recap/take home from each "tool" section (such as line 180) might help the reader to grasp what is essential. I suggestion is to summarize the main findings, and challenges in the end of R&D or in the conclusion section.

line 152-153 Is RNA.seq data available for this strain? What is defining "such regions"?

line 197 line 212, 236 Since it is stated that PMQAK1 gives a high number of false positives. The authors need to explain in the text why they continued to use this plasmid for promoter and terminator characterization. There are a number of other shuttle vectors available for the model cyanobacterium Synechocystis that could have been tested.

line 200 What promoters were used to regulate eYFP and Cas12a expression? Please specify.

line 222 Are the limited number of analyzed systems enough to support this conclusion? Please discuss.

Line 254 The chromosomal copy number would have been useful for evaluation of the results as well as to strengthen the discussion. Instability of this vector in PCC11901 would give similar results. Or was this examined?

Figure 3. It is well known that the expression level form a specific promoter depends on the sequence of the ORF expressed. And that promoter strength could not be decided by using merely FP genes such as YFP as test system. This is neglected by the authors, and should to be discussed in this manuscript, and optionally validated by using other genes.

line 384 This reviewer finds it very strange that the promoters analyzed as leaky are used for Cas9 expression. Please clarify why these promoters were used.

line 409 and onward, qPCR results are needed with CRISPRi to show that the phenotypic effect observed is in fact related to a decreased transcript number of the target, and not for example CRISPR toxicity. In particular Supplementary Figure 5 on page 49, the induced sample on the SDS page (panel A) has much fainter bands in general than the WT and uninduced samples. Proteins unrelated to their CRISPRi targets also seem less abundant, which questions whether the fainter bands of cpcB and nblA (and the associated phenotypic changes) are in fact caused by transcriptional downregulation.

line 494 The strong conclusion is not supported by the results. For this general conclusion a number or additional essential genes needs to be targeted.
Especially since the polyploidy of this strain is unknown.

line 511 What is the advantage of having this "hybrid suicide vector" compared to having both the gRNA and repair cassette onto the same self-replicating vector than Cas12a? It seems like efficiency would be decreased since it requires two separate double crossover events.

line 547 It would be good to have a uninduced control in this case to quantify spontaneous HR events that are not mediated by Cas12.

Line 561 Any reason why the second knock-in has a much lower editing efficiency in the iterative genome editing?

Conclusion, Please provide a conclusion specific to the interesting results and discussion in the manuscript. As it reads now it is very general and don´t enhance the impact of the manuscript. One suggestion is to conclude the novel tools and maybe highlight where already well-developed genetic tools for model cyanobacteria can be used.

Response to reviewers

We are very glad to see that both reviewers found interest in our work and wish to thank them for the time and effort put into the review process. We do feel this has helped to further improve the quality of our manuscript.

Reviewer #1

In the research article under review, authors describe the toolbox development for one of the recent, high biomass generating, marine cyanobacterial strain Synechococcus sp. PCC 11901. The authors have performed detailed characterization of different promoters, their regulation, antibiotic resistances, neutral sites, etc. for leveraging this system as a platform cell factory for production of different chemicals. While the article meticulously describes about these developments and present a number of helpful findings, there are some necessary revisions before considering for the publication.

We thank the reviewer for their kind comments.

Comments:

1. Title: The manuscript title is very broad, whereas the paper is specifically directed towards toolbox development. Authors can rephrase the title to specifically convey the content of the paper.

We agree and have now modified the title to ‘A toolbox to engineer the highly productive cyanobacterium *Synechococcus* sp. PCC 11901’.

2. Neutral sits for

This comment was incomplete.

3. Introduction: a) Authors can try to refer to more recent publications (2020-2023) and be precise with the same. For instance, line 52-53 in the introduction mentions about PCC 6803 and PCC 7942; but the first reference for biofuels (Kopka et al., 2017) is based on PCC 7002. Authors can either include PCC 7002 as an organism and/ or include references, https://doi.org/10.1002/bit.28479, https://doi.org/10.1016/j.biortech.2021.125852.

Thank you for picking this up. We have now modified line the sentence on line 53: ‘Unicellular model cyanobacterial strains, such as *Synechocystis* sp. PCC 6803 (hereafter PCC 6803), *Synechococcus* elongatus PCC 7942 (hereafter PCC 7942) and *Synechococcus* sp. PCC 7002 (hereafter PCC 7002) have been investigated as biorefineries for bulk commodity products such as biofuels (Kopka et al., 2017; Sawant et al., 2021), bioplastics (Khetkorn et al., 2016), natural food additives (Puzorjov et al., 2022; Pritam et al., 2023)…’

b) Line 58: When authors mention slow growth rate, it will be good to mention '...as compared to...', because cyanobacteria have faster growth rate among photoautotrophs, but with less biomass generation.

On line 61 we have added: “compared to heterotrophic chassis”.

c) Line 73: Authors refer to the cell densities of E coli as 24.7 g/l. This needs correction and a more appropriate reference. Cell densities of 100-150 g DCW/lit are routinely obtained for E coli. We apologise for this error. We have modified the sentence and used a more recent and appropriate reference from the Wangikar lab (Ganjave et al., 2022; https://doi.org/10.1016/j.btre.2021.e00694) that discusses biomass yields in *E. coli* cultured in shake flasks. The sentence on line 73 now reads: ‘…PCC 11901 has an additional capacity for sustained growth to high densities (up to 30 g/L dry cell weight), similar to the biomass accumulation observed for fed-batch-cultured *E. coli* in shake flasks (Ganjave et al., 2022).’

d) High cell densities for cyanobacteria have been reported previously as well. E.g. Alagesan et al 2013 Bioresource Technology

Alagesan et al. (2013) obtained a maximum biomass of 12.8 g/L, and only under mixotrophic conditions, a strategy which is challenging to implement at large scale due to contamination issues. In contrast, higher cell densities are obtained with PCC 11901 under photoautotrophic growth. Thus, we would argue that comparison in this case is not appropriate.

e) Lines 142-143: Additional neutral sites have been reported by PCC 11801 by Madhu et al 2023 ACS Synthetic Biol. Comparison to those may be useful.

We have now expanded the sentence on line 135: 'Several neutral sites have been identified in model cyanobacterial species…and the new fast-growing strain PCC 11801 (Madhu et al., 2023).' Unfortunately, a comparison with this study was not feasible. Madhu et al. (2023) only report linear growth rates and did not provide data on complete growth curves. In contrast, our study reports growth of the WT and neutral sites (NS) mutants to a high OD (Figure 1C). We observed that all NS mutants showed similar linear growth rates at low OD (OD < 20), but significantly different final ODs. We believe that this is a key result when choosing NSs for biotechnology applications.

4. Results and discussion:

a) I did not find any mention about how stable the transformed cultures are. Metabolically engineered strains of cyanobacteria are notoriously unstable and tend to revert.

We disagree here in terms of transformant strains with genome-integrated DNA that have been fully segregated. Between the Lea-Smith and McCormick labs, we have generated hundreds of mutants and reversion has not been an issue as long as the strains have been properly segregated to homoplasmy following transformation. We routinely check strains, especially unmarked mutants, by PCR months and years after generation and have never observed reversion. As an example from PCC 11901, the Δ*mrr*::eYFP strain was made in 2021 and still retains a stable inserted YFP cassette at the *mrr* neutral site.

b) Authors need to check the entire manuscript for any typo-errors. For instance, line 117 has PC11901 instead of PCC 11901.

Thank you for picking this up. We corrected this now on line 119 and rechecked the manuscript for typo-errors.

c) Figures:

a) In figure 1, giving the pictures of the colonies on the plates look feasible, but does not convey any significant results to the reader. Authors can think of moving it to the supplementary information or remove it completely.

We would humbly ask to keep these images as illustrations for readers not familiar with cyanobacterial research to show what colonies might look like if they attempt transformation themselves. However, if the editorial team feels they would be best removed we would be happy to do so.

b) In figure 1, the term 'vector' is used in the first column, while the third column indicates transformants per ug of 'plasmid'. Although the two jargons can be used interchangeably, they are not the same. So it would be good to be consistent.

Thank you for spotting this, column 3 has now been modified to 'Transformants per µg of vector'. Where appropriate, we have also modified 'plasmid' to 'vector' throughout the manuscript for improved consistency.

c) There are multiple sub-figures in figures 2, 3, 5, 6 that lack the X-axis label. E.g. 2F, 3A/B/D, 4B/C/D/E, etc.

These are bar graphs that have sample names shown on the x-axis. We humbly disagree that additional x-axes are required here. However, we would be happy to include these if the editorial team request them.

d) Authors have separately labelled each sub-figure within figure 5. It would be good to club certain figures, because independently, they are unable to convey any result. E.g. F-G, J-K, etc. Secondly, the figure legends mentioned in H, are assumed to be applicable for everything thereafter. However, it needs to be specifically mentioned in the caption or presented as a common legend at the bottom of the four figures.
We thank the reviewer for these suggestions. We have now aggregated Fig 5F-I (now F and G) and J-M (now H and I). In line with the request for RT-qPCR analyses from reviewer 2, we have moved the growth curves (previously H and L) to Fig. S6, and replaced them with RT-qPCR results. The legend text has been modified for the new format. We have clarified that 5F-I utilise the PhlF/PphlF CRISPRi-dCas9 system.

Reviewer #2 (Comments for the Author):
The manuscript focus on identifying and characterising genetic tools for a cyanobacterium that is fast growing and of interest as a host for biotechnological applications. There is a lack of synthetic biology parts and tools for engineering this photosynthetic microbe efficiently. To this reviewer the objective of this manuscript is dual, first to characterise, analyse and evaluate different tools and second to recommend a valuable tool-box. Maybe the manuscript would benefit from being more distinct about when either is discussed. The work presented are of high quality and a number of useful tools have been developed and characterized. The manuscript is very clearly written and the figures are informative with smart design and add to the manuscript. The work is sound and all methods and strategies used are in general appropriate for the study. Novel and potentially very useful results are presented. We have a few major concerns with this manuscript. These are communicated to the authors and should be addressed. Some additional experiments are strongly suggested for the work to be as useful as stated in the conclusion.
We thank the reviewer for their endorsement and positive feedback.

RESULT AND DISCUSSION
General: The Result and Discussion part is overwhelming, maybe too detailed and some of the important results are masked by much less important information/text. A more focused R&D would be beneficial and a short recap/take home from each "tool" section (such as line 180) might help the reader to grasp what is essential. I suggestion is to summarize the main findings, and challenges in the end of R&D or in the conclusion section.
We thank the reviewer for this critique. Our intention was for each Results and Discussion sub-section to be focused on a specific tool component, and for each to be self-contained and thorough in terms of literary context. We feel it is important to include some discussion and review, and have checked that each sub-section contains a suitable summary. In line with a later comment (below), we have also modified the conclusion section from line 619 to be more focused on the tools developed and their applications.

line 152-153 Is RNA.seq data available for this strain? What is defining "such regions"?
(*from the pdf*) How do the authors know this is not coding for regulatory elements?
Unfortunately, there is no published RNAseq data available for PCC 11901 yet (although we agree this would be useful). We do not know if the NS1 locus contains regulatory elements. We hypothesised this putative intergenic region was a potential neutral site due to its location between two hypothetical protein coding sequences (FEK30_11550 and FEK30_11555) that face each other.

We screened the 185 bp region for regulatory elements (e.g. promoter and terminators) using online tools, but none were identified. Ultimately, the NS1 mutant showed slower growth at higher densities and a lower final OD than WT (Figure 1C), which suggested that this is not an optimal site for genomic integrations and that there may be one or more regulatory elements associated with this site.

line 197 line 212, 236 Since it is stated that PMQAK1 gives a high number of false positives. The authors need to explain in the text why they continued to use this plasmid for promoter and terminator characterization. There are a number of other shuttle vectors available for the model cyanobacterium Synechocystis that could have been tested.
(*from the pdf*) Why carry subsequent work with pPMQAK1 plasmids when false-positives (and potentially higher plasmid instability) with this plasmid have been observed?
Thank you for highlighting this omission. We observed that transconjugant colonies that retained a dark green phenotype after the first re-streak were stable on kanamycin and retained the PMQAK1 vector. Thus, the PMQAK1 plasmids were still suitable for promoter and terminator analyses. We have included a stability experiment for transconjugants carrying the pPMQAK1 vector (Supplementary Figure S3) and modified the sentence on line 198: 'Colonies with a dark green phenotype remained viable on kanamycin and could retain the pPMQAK1 vector when cultured for up to 30 days, even in the absence of selection (**Supplementary Figure S3**), suggesting that RSF1010-based vectors are retained for several generations in PCC 11901, as observed in PCC 6803 (Nagy et al., 2021; Puzorjov et al., 2022).'

We agree that testing other shuttle vectors would be of significant interest, but we feel is outside of the scope of this study. Our focus here was to test the amenability of PCC 11901 to transconjugation and to outline a robust selection system.

line 200 What promoters were used to regulate eYFP and Cas12a expression? Please specify.
We used the Pcpc560 promoter and TrrnB terminator for both. This information is now included in Supplementary Table S2. On line 208 we have now included a reference to Supplementary Table S2.

Line 207
(*from the pdf*) What about vector stability?
We have address this above.

Line 221
(*from the pdf*) Did the authors perform any experiment on plasmid stability?
We have address this above.

line 222 Are the limited number of analyzed systems enough to support this conclusion? Please discuss. (*from the pdf*) This is a very general conclusion for such a small number of target sites.
We agree that other self-replicating vectors may perform better. We have added an additional sentence on line 231: 'Furthermore, other self-replicating vector systems may perform better than the RSF1010-based vectors tested here (e.g. Liu & Pakrasi, 2018; Opel et al., 2022).'

Line 252
(*from the pdf*) Some promoters seem to behave differently when integrated vs on plasmid. Could this be linked to plasmid stability?
We have addressed stability above. We found the trend in promoter expression to be very similar between transformants and transconjugants (Figure 3C; $R^2 = 0.95$).

Line 254 The chromosomal copy number would have been useful for evaluation of the results as well as to strengthen the discussion. Instability of this vector in PCC11901 would give similar results. Or was this examined?
We agree that determining chromosomal copy number would be very interesting. However, such work would constitute an entirely new study as chromosomal copy number is known to alter depending on culture conditions, i.e. changes in light and nutrient levels, temperature and stages of growth (Zerulla et al., 2016 Microbiology, https://doi.org/10.1099/mic.0.000264; Pope et al., 2020 Front Plant Sci https://doi.org/10.3389/fpls.2020.00372). We are keen to explore this in future work but feel that it is outside of the scope of the current study.

Figure 3. It is well known that the expression level form a specific promoter depends on the sequence of the ORF expressed. And that promoter strength could not be decided by using merely FP genes such as YFP as test system. This is neglected by the authors, and should to be discussed in this manuscript, and optionally validated by using other genes.
We agree that the ORF has an impact on expression from a specific promoter. Our goal here was to provide a broad screen of promoter strengths based on a common standard (i.e. YFP) used for characterising promoter libraries. We have included the following sentence on line 242: 'It should be noted that eYFP expression is a proxy of promoter strength, and that expression levels can vary depending on the sequence of the open reading frame.'

line 384 This reviewer finds it very strange that the promoters analyzed as leaky are used for Cas9 expression. Please clarify why these promoters were used.
We were interested in characterising the impact of inducible promoter leakiness on dCas9 activity, so we compared the RhaS/P*rhaBAD* and P*trcE** systems to PhlF/P*phlF*. We felt that this was important to demonstrate and inform other inducible CRISPR work. After confirming that the PhlF/P*phlF* system was tightly regulated, we only progressed with the latter (line 416 onward).

line 409 and onward, qPCR results are needed with CRISPRi to show that the phenotypic effect observed is in fact related to a decreased transcript number of the target, and not for example CRISPR toxicity. In particular Supplementary Figure 5 on page 49, the induced sample on the SDS page (panel A) has much fainter bands in general than the WT and uninduced samples. Proteins unrelated to their CRISPRi targets also seem less abundant, which questions whether the fainter bands of cpcB and nblA (and the associated phenotypic changes) are in fact caused by transcriptional downregulation.
We solidly agree. We have performed RT-qPCR experiments for work in Figure 5 on CRISPRi repression of *cpcB* and *nblA*, and repeated the Coomassie-stained SDS-PAGE gel analysis in Supplementary Figure S6 with more controls. Our results now clearly show that dCas9 alone (induced or uninduced) has no apparent impact on growth, visible phenotype or protein abundance. Only induction of dCas9 in the presence of the sgRNA resulted in a phenotype, which was linked to a significant reduction in *cpcB* and *nblA* transcript abundances (now Figure 5F and 5H). For CRISPRi of *cpcB*, the new protein gel shows that CpcB and CpcA were much reduced (Supplementary Figure S6B), but there was no apparent impact on other proteins in the sample, which we feel now demonstrates the lack of off-target effects. We have modified the text in the three paragraphs from line 416 to reflect these findings.

line 494 The strong conclusion is not supported by the results. For this general conclusion a number or additional essential genes needs to be targeted. Especially since the polyploidy of this strain is unknown.
We did test the two sites that were successfully utilised in the closely related species, PCC 7002, by Jones et al. (2021). Therefore, we are doubtful that any other essential genes will work. However, we

recognise the point of the reviewer and have modified the sentence on line 502: ‘Although other essential genes could be trialled, our inability to generate markerless mutants…’.

line 511 What is the advantage of having this "hybrid suicide vector" compared to having both the gRNA and repair cassette onto the same self-replicating vector than Cas12a? It seems like efficiency would be decreased since it requires two separate double crossover events.
We feel our system has several advantages over an ‘all in one’ approach. First and foremost, the modular design allows for (unlimited rounds of) iterative CRISPR engineering without the need to cure the strain from the existing RSF1010 editing vector. It is also generally easier to perform natural transformation rather than transconjugation for every edit, thereby streamlining the process. Second, our design separates genomic integration from Cas12a activation. As we demonstrated in Figure 6B, this allows more time for homologous recombination to occur prior to Cas12a activation, and improves efficiency significantly. Lastly, the many colonies obtained on the selective plate after plating indicates that the second homologous recombination event (i.e. the AbR cassette swap/sgRNA delivery to the editing vector) likely does not impact efficiency significantly, while also providing a good control for transformation.

line 547 It would be good to have a uninduced control in this case to quantify spontaneous HR events that are not mediated by Cas12.
We did include an uninduced control - on line 543: ‘In the absence of DAPG, no edits were observed in the colonies that grew, indicating that Cas12a expression was tightly repressed.’

Line 561 Any reason why the second knock-in has a much lower editing efficiency in the iterative genome editing?
We are unsure why we obtained a 56% efficiency for mCherry insertion into the aquI neutral site in the WT strain, and 31% into the mrr::eYFP strain for iterative CRISPR. We suspect that this may be biological and/or experimental variation. However, we are still pleased with these reasonably high success rates and feel they will facilitate use of the approach.

Line 606 Conclusion, Please provide a conclusion specific to the interesting results and discussion in the manuscript. As it reads now it is very general and don´t enhance the impact of the manuscript. One suggestion is to conclude the novel tools and maybe highlight where already well-developed genetic tools for model cyanobacteria can be used.
As outlined in the first comment, we have now also modified the conclusion section from line 619 to be more focused on the tools developed and their applications.

Figure 1
(*from the pdf*) Is part of the neutral site deleted or the gene is only disrupted?
We have included the full sequences of the flanks for the neutral sites in Supplementary Data S1.
In brief:

- The CDS for mrr (FEK30_09380) is completely removed.
- The CDS for glgA1 (FEK30_14880) is completely removed.
- 853 bp of the CDS (1053 bp) of desB (FEK30_04840) is removed.
- 909 bp of the CDS (954 bp) of aquI (FEK30_10065) is removed.
- The intergenic region (185 bp) for NS1 (between FEK30_11550and FEK30_11555) is removed.

Figure 3
(*from the pdf*) What is the background fluorescence? This needs an empty vector or promoterless YFP control.

Background fluorescence of the wild-type PCC 11901 strain (for transformants) and an empty vector control (for transconjugants) was subtracted from the values shown. The background fluorescence of these lines was significantly lower than the expression lines (~700 AU).

Figure S5
(*from the pdf*) In general the induced sample has much fainter bands than the WT and uninduced samples... This would be a much stronger dataset with qPCR results.
We have address this above.

14-Apr-2024
Prof. Alistair J McCormick
The University of Edinburgh School of Biological Sciences
School of Biological Sciences
Edinburgh EH9 3BF
United Kingdom

MSID: PP2024-RA-00507D
MS TITLE: A toolbox to engineer the highly productive cyanobacterium Synechococcus sp. PCC 11901

Dear Dr. McCormick:

I am pleased to inform you that your revised manuscript "A toolbox to engineer the highly productive cyanobacterium Synechococcus sp. PCC 11901" has been accepted for publication in Plant Physiology.

I have recommended your paper for highlighting with a News and Views article; expert reviewers saw it as a substantial technical advance, and cyanobacteria are, of course, high-profile and *plant-adjacent* synthetic biology platform organisms.

Your manuscript will be checked for consistency with journal formatting and image standards by one of our Science Editors, who will contact you shortly. They may have a number of queries and suggestions for you. Please keep in mind that they work to increase the online visibility of your article and its attraction for readers from the widest of backgrounds. You will have the opportunity to review any revisions, and I hope you will work constructively with them to get the most out of your research. You will also have the opportunity to review all changes when you receive the author proofs for your article.

Once validated for publication, your article will be sent to a Science Editor for a final review of your manuscript and source files. After your final files are uploaded, your article will appear in the next available issue and the online Preview version of your article will be posted after your signed Copyright and Checklist forms and final source files are received.

Please be sure to include the following in your manuscript file:
Harvard style citations
List of all author contributions and funding information at the beginning of manuscript after the titles and author list.
List all supplemental materials associated with the article, with titles, at the end of the ms, before the references and acknowledgments section.
Complete Figure Legends for all figures appearing in the article listed before the Literature Cited.

Thank you and congratulations on your Plant Physiology paper!

Sincerely, Board of Editors, Plant Physiology

=====
FIRST AUTHOR PROFILES
=====

We recognize the hard work that goes into being First Author of a Plant Physiology paper by publishing a "First Author Feature" for each First Author alongside your paper.

If the first author(s) would like to submit information for a feature on Facebook and Twitter, fill out the form by clicking on the link below:

Plant Physiology First Author Feature Form: https://forms.office.com/r/ZBCGZfhm8F
If you cannot access the form through the link above, please contact Rachel at rbelsky@aspb.org.

To upload an image of yourself to accompany your profile, please put your photo in the correct journal folder at the link below. You must label your image: Author First Name Last Name Article Title.

First Author Profile Image Dropbox: https://www.dropbox.com/scl/fo/xz029p7qjr2jml2rp2kmq/h?

dl=0&rlkey=6fkxcaqmxx35ap152b2osy1fz

=====
IMPORTANT REMINDER: PEER REVIEW REPORTS
=====

If you opted to publish a peer review report along with your article during the original submission process, it will be prepared by the editorial staff and publicly posted with your manuscript, inside the zip file that contains any other supplemental material. As a reminder, the peer review report is a public record of all comments from editors and reviewers, as well as your prior responses, as you received them in the decision letters for each draft of your manuscript. If you agreed to publish this report and have changed your mind, or are not sure if you selected this option, please contact the editorial office as soon as possible before signing the license agreement from our publisher.

-- Reviewer comments:
Reviewer #2 (Comments for the Author):

The authors have addressed all comments to our review. We are satisfied with the answers and revisions made.
